# Methyl Chloride for Local Anæsthesia

**Published:** 1889-05

**Authors:** B. Ward Richardson


ARTICLE VIII.

### METHYL CHLORIDE FOR LOCAL ANÆSTHESIA.

BY DR. B. WARD RICHARDSON.

When chloride of methyl is condensed, it produces, on being liberated through a jet, an intense cold. It has therefore been used commercially for refrigerating the carcasses of dead animals intended for consumption as food. This use of it has lead to the suggestion of employing it for freezing parts of the living body in the same manner as ether spray is employed for local anæsthesia. The idea of such use occurred to me soon after I introduced ether spray, but was abandoned because of the difficulty of managing the gas for the purpose required. The difficulty lies in so regulating the escape of the gas, as to be sure of doing enough without doing too much.

In itself the chloride of methyl has no local anæsthetic properties, as some ignorant notices of it in the press would lead the reader to believe. It acts simply and solely like ether spray, by the cold it induces, and the surgeon who would employ it should fully understand that he is simply substituting one cold producer for another. As a matter of practise, ether or rhigolene spray for benumbing with cold is in every sense the readier and more practical method, and the principle at work is the same.

For causing local anæsthesia by extreme cold compressed nitrous oxide, carbonic acid, or sulphurous acid act in a similar manner to compressed methyl chloride. I once pursued a line of enquiry for local anæsthesia with nitrous oxide and with carbonic acid, the jet of each of which produced local anæsthesia. A jet of the nitrous gas playing upon the skin destroyed sensibility immediately by the cold it caused, and once acted with such intensity that a burn was the result. A workman, was engaged in fitting up a nitrous oxide apparatus in my laboratory, brought his arm accidentally across a jet of the gas escaping at great pressure, and was so severely "chill-burned" that I had to treat him as for a burn from heat. This man told me that the accident was not uncommon amongst his class of workmen, and that they called the wounds that followed exposure "*chill burns.*"

After seeing the action of these gases as above described, it occurred to me that they might be used as cauteries for the removal from the skin of warts and small pendulous growths. Their jet is much less alarming than either the heated wire or the knife. It is also very rapid in its action, and at the moment painless, although, as a matter of course, there is some pain and soreness afterwards.

Here is a new line of practice for surgeons, for removal of foreign growths by *the freezing cautery*, a practice which I do not for a moment doubt they will one day take up and follow out to important results. There are other gases beside those named which might be used. Compressed chlorine, delivered in a very fine jet, would be magically destructive. Combinations of true anæsthetics and freezing jets might also easily be obtained, under which large portions of the body could be painlessly removed.

In commencing this line of practice there can be no doubt as to the best gas to commence with. The best gas is carbonic acid. It is cheaply and readily made; it is easily condensed, admitting even of being condensed into the solid form, but on being set free as spray from the liquid state it causes sufficient refrigeration. One great advantage of it is that, unlike methyl chloride, it is not inflammable, and can be used, therefore, with artificial light. It is also slightly anæsthetic.—*The Asclepiad.*

